# Ranking the Predictive Power of Clinical and Biological Features Associated With Disease Progression in Huntington's Disease

**DOI:** 10.3389/fneur.2021.678484

**Published:** 2021-05-20

**Authors:** Naghmeh Ghazaleh, Richard Houghton, Giuseppe Palermo, Scott A. Schobel, Peter A. Wijeratne, Jeffrey D. Long

**Affiliations:** ^1^F. Hoffmann-La Roche Ltd., Basel, Switzerland; ^2^Department of Computer Science, Centre for Medical Imaging Computing, University College London, London, United Kingdom; ^3^Department of Neurodegenerative Disease, Huntington's Disease Research Centre, Queen Square Institute of Neurology, University College London, London, United Kingdom; ^4^Department of Psychiatry, University of Iowa, Iowa City, IA, United States; ^5^Department of Biostatistics, University of Iowa, Iowa City, IA, United States

**Keywords:** Huntington's disease, disease progression, prognostic variables, machine learning, random forest

## Abstract

Huntington's disease (HD) is characterised by a triad of cognitive, behavioural, and motor symptoms which lead to functional decline and loss of independence. With potential disease-modifying therapies in development, there is interest in accurately measuring HD progression and characterising prognostic variables to improve efficiency of clinical trials. Using the large, prospective Enroll-HD cohort, we investigated the relative contribution and ranking of potential prognostic variables in patients with manifest HD. A random forest regression model was trained to predict change of clinical outcomes based on the variables, which were ranked based on their contribution to the prediction. The highest-ranked variables included novel predictors of progression—being accompanied at clinical visit, cognitive impairment, age at diagnosis and tetrabenazine or antipsychotics use—in addition to established predictors, cytosine adenine guanine (CAG) repeat length and CAG-age product. The novel prognostic variables improved the ability of the model to predict clinical outcomes and may be candidates for statistical control in HD clinical studies.

## Introduction

Huntington's disease (HD) is a rare, genetic, neurodegenerative disease caused by a cytosine adenine guanine (CAG) repeat expansion variant of the huntingtin gene (*HTT*) (1) and is characterised by a triad of cognitive, behavioural, and motor symptoms (2, 3). Disease onset, defined as the onset of motor signs and symptoms as measured by a Diagnostic Confidence Level of 4 (3, 4), typically occurs in the prime of life, between the ages of 30 and 50 years (2). HD is associated with increasing disability, worsening of function and loss of independence, leading to death within approximately 15 years of onset (2, 5). Motor and cognitive symptoms deteriorate steadily as the disease progresses (3, 6–9), while behavioural symptoms tend to be episodic (10).

With potential disease-modifying therapies for HD in clinical development (11), there is interest in measuring disease progression and characterising prognostic variables in order to improve the efficiency and accuracy of clinical trials (12). Prognostic variables can be used to identify a patient population through an enrichment strategy to reduce interpatient variability in clinical trials or alternatively to enrich for faster progressors, and to eventually inform the optimum time to start treatment (12). Statistically controlling for prognostic baseline variables may also be important in non-randomised (e.g., open-label) studies as they could confound the relationship between treatment exposure and outcomes. Additionally, when testing hypotheses in randomised studies, the probability of detecting a treatment effect will usually increase by including prognostic variables as covariates in the analysis, as this would explain a significant amount of variability observed due to random error.

Large prospective cohort studies have shown that manifestations of progression, that is, clinical signs and symptoms of HD, as well as known biological predictors of progression such as CAG repeat length and CAG-age product (CAP) score, can predict clinical progression or motor onset (7, 13). However, no study has systematically ranked the importance of predictors of progression in a manifest HD population (i.e., after the onset of unequivocal motor symptoms).

Random forest (RF) regression models permit interrogation of large, complex clinical datasets to capture non-linear associations between multidimensional predictive variables and clinical outcomes with high predictive accuracy (14, 15). RF approaches are well-suited to classification and regression problems, such as identifying variables with predictive potential for disease progression from clinical datasets. Here, we use modern machine learning methods to examine a large number of HD variables to identify the most important predictors of progression on five clinical outcomes: total functional capacity (TFC), a measure of function; stroop word reading (SWR), a measure of attention and psychomotor processing speed; symbol digit modalities test (SDMT), a measure of executive function, visuo-spatial working memory, attention and processing speed; total motor score (TMS), a measure of motor function; and the composite unified HD rating scale (cUHDRS), an equally weighted composite outcome measure of the TFC, TMS, SDMT, and SWR that was developed based on an early manifest HD population (16). The large prospective Enroll-HD cohort (NCT01574053) is used to investigate the relative contribution and ranking of potential prognostic variables to predict clinical progression in a clinical trial-like manifest HD population.

## Results

The analysis included 1,608 individuals meeting typical criteria for clinical trials in manifest HD and with CAG repeats between 36 and 64 (filtering criteria shown in Table 1). Patient demographics are shown in Table 2.

**Table 1:** Attrition table showing number of patients included after applying filters for each inclusion criterium.

**Total initial population in the dataset: 15,301**
1) Age at baseline = 25–65 years; diagnosis age >20 years: **6,432**
2) Manifest only (HDCAT = 3): **6,025**
3) DCL = 4: **5,694**
4) IS at baseline 100 ≥ IS > 70: **4,277**
5) At least 2 year's follow-up score for all four outcomes: **1,608**

*Totals are for subjects with complete data on all outcome scores*.

*DCL, diagnostic confidence level; HDCAT, HD category; IS, Independence Scale*.

*The bold values indicate the number of patients included after applying each inclusion criterium*.

**Table 2:** Patient demographics.

**Demographic**	***N* = 1,608**
Age, years, mean (SD)	49.60 (9.32)
Male sex, *n* (%)	819 (50.9)
**Region**, ***n*** **(%)**
Australasia	69 (4.3)
Europe	1,068 (66.4)
Latin America	9 (0.6)
North America	462 (28.7)
CAG repeat length, mean (SD)	43.93 (3.04)
CAP score, mean (%)	488.18 (82.35)
Shoulsen and Fahn Stage at baseline, *n* (%)	Stage I: 716 (44.53) Stage II: 731 (45.49) Stage III: 159 (9.89) Stage IIII: 2 (0.12)

*CAG, cytosine adenine guanine; CAP, CAG-age product; SD, standard deviation*.

**Figure 1:**
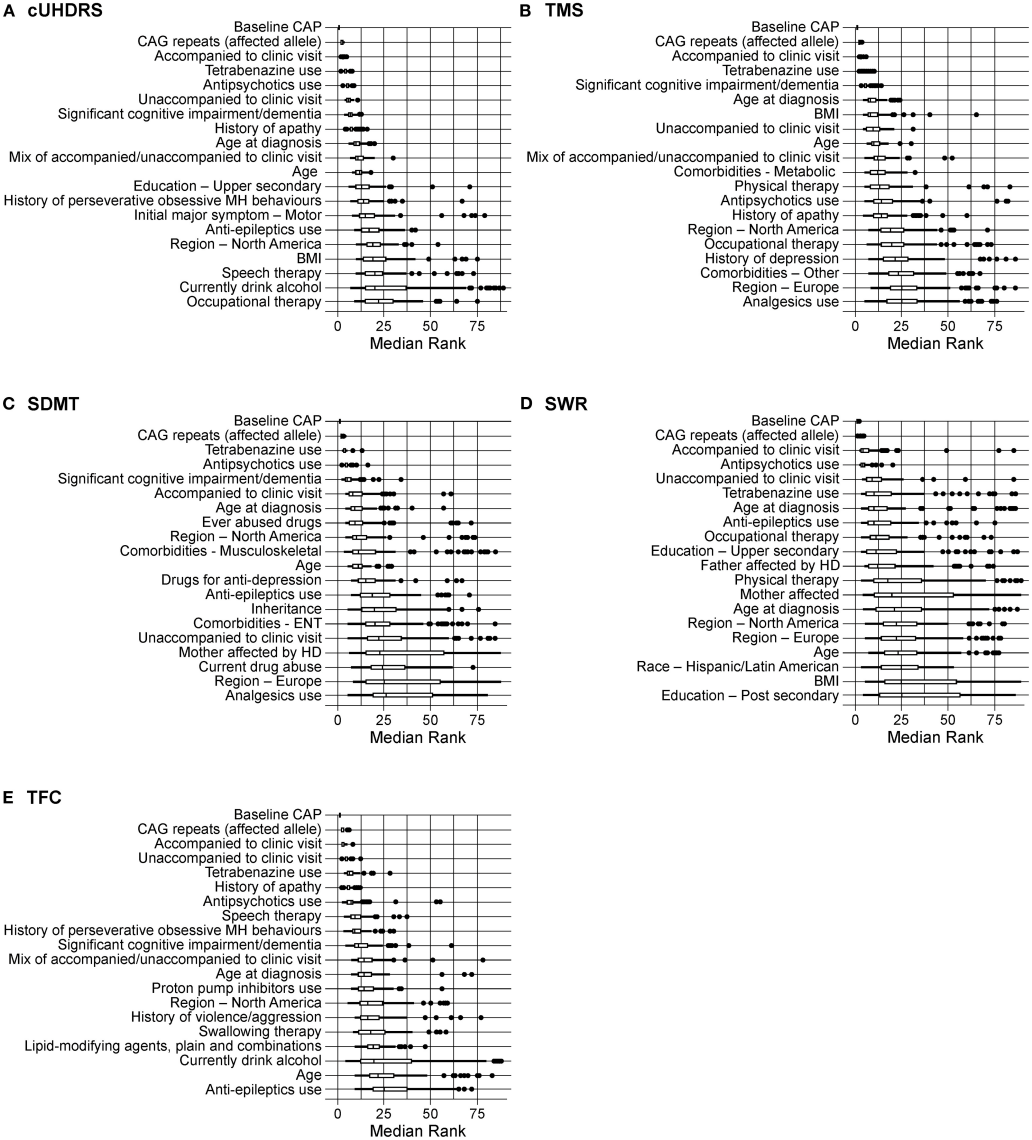
Rankings of predictors of clinical progression as measured by **(A)** cUHDRS; **(B)** TMS; **(C)** SDMT; **(D)** SWR; **(E)** TFC. Boxplots are shown with the upper box edge representing the 75^th^ quantile and the “whisker” extending to 1.5 times the IQR. A circle is an outlier, defined as a ranking that extends beyond a whisker. BMI, body mass index; CAG, cytosine adenine guanine; CAP, CAG-age product; cUHDRS, composite Unified HD Rating Scale; ENT, ear, nose, throat; IQR, interquartile range; MH, mental health; SDMT, symbol digit modalities test; SWR, stroop word reading; TFC, total functional capacity; TMS, total motor score.

**Figure 2:**
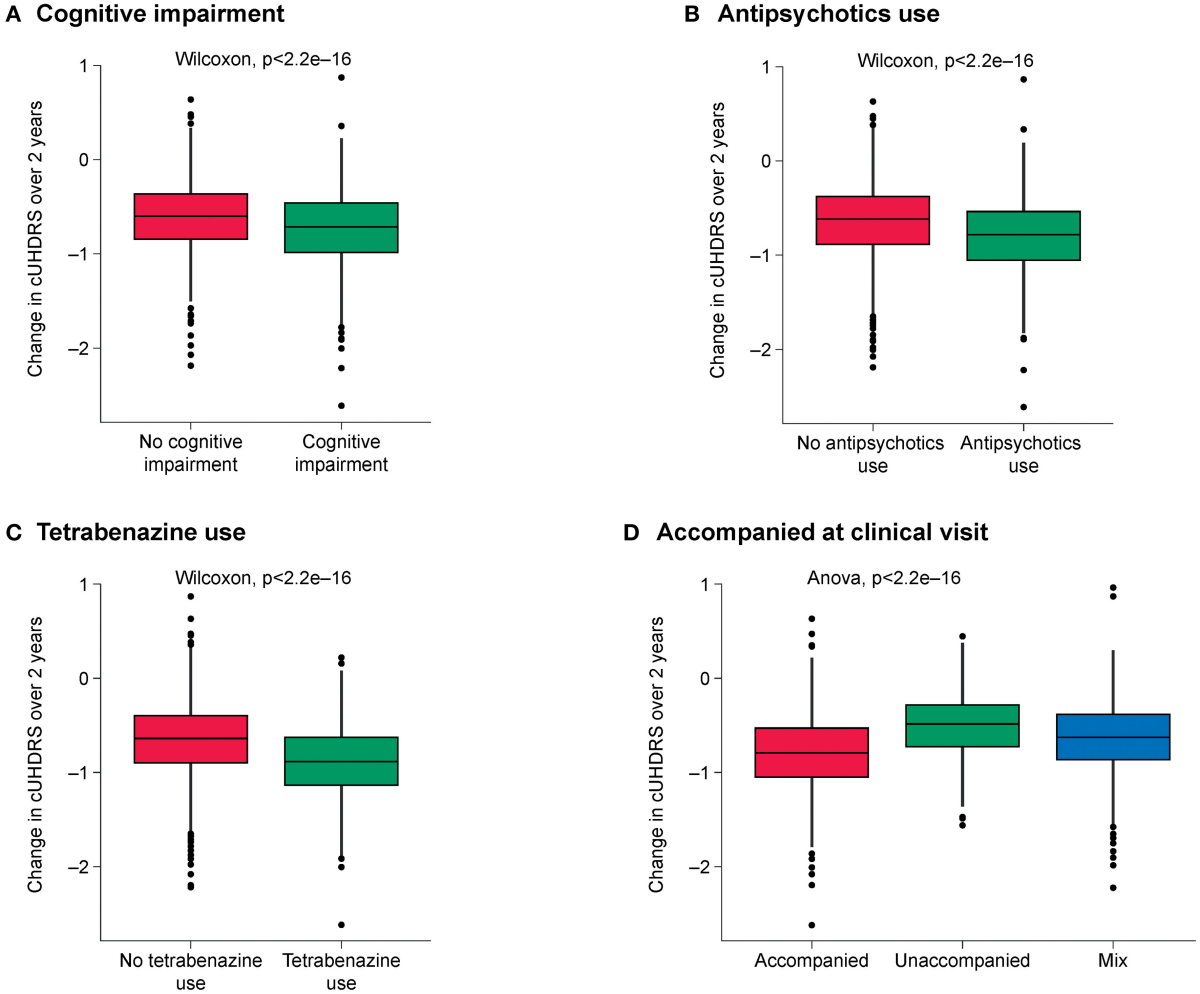
Effect of the highest-ranked variables on clinical progression trajectory as measured by cUHDRS. **(A)** Cognitive impairment; **(B)** Antipsychotics use; **(C)** Tetrabenazine use; **(D)** Being accompanied at clinic visit. cUHDRS, composite Unified HD Rating Scale.

The highest-ranked variables predictive of disease progression for each outcome are shown in Figure 1 and the top 10 variables for each outcome shown in Table 3. CAP was found to be the most predictive variable for all outcomes and CAG repeat length was ranked as the second most important variable for all outcomes. Other prognostic variables associated with faster progression that ranked in the top 10 for at least three of the five outcomes were: age at diagnosis (all but SWR and TFC), being accompanied to clinic visits (for all outcomes), history of cognitive impairment (all but SWR), tetrabenazine use (for all outcomes) and antipsychotics use (all but TMS). The effect of these variables on disease progression trajectory as measured by the cUHDRS is shown in Figure 2.

**Table 3:** Top 10 predictive variables for each outcome.

**Rank**	**Outcome**				
	**cUHDRS**	**TMS**	**TFC**	**SDMT**	**SWR**
1	Baseline CAP	Baseline CAP	Baseline CAP	Baseline CAP	Baseline CAP
2	CAG repeats (affected allele)	CAG repeats (affected allele)	CAG repeats (affected allele)	CAG repeats (affected allele)	CAG repeats (affected allele)
3	Accompanied to clinical visit	Accompanied to clinical visit	Accompanied to clinical visit	Tetrabenazine use	Accompanied to clinical visit
4	Tetrabenazine use	Tetrabenazine use	Unaccompanied to clinical visit	Antipsychotics use	Antipsychotics use
5	Antipsychotics use	Significant cognitive impairment or dementia	Tetrabenazine use	Significant cognitive impairment or dementia	Unaccompanied to clinical visit
6	Unaccompanied to clinical visit	Age at diagnosis	History of apathy	Accompanied to clinical visit	Tetrabenazine use
7	Significant cognitive impairment or dementia	BMI	Antipsychotics use	Age at diagnosis	Age at diagnosis
8	History of apathy	Unaccompanied to clinical visit	Speech therapy	History of drug abuse	Anti-epileptics use
9	Age at diagnosis	Age	History of perseverative obsessive MH behaviours	Region—North America	Occupational therapy
10	Mix of accompanied and unaccompanied to clinical visit	Mix of accompanied and unaccompanied to clinical visit	Significant cognitive impairment or dementia	Comorbidities—musculoskeletal	Education—upper secondary

*BMI, body mass index; CAG, cytosine adenine guanine; CAP, CAG-age product; cUHDRS, composite Unified HD Rating Scale; MH, mental health; SDMT, symbol digit modalities test; SWR, stroop word reading; TFC, total functional capacity; TMS, total motor score*.

The common variables among the top 10 most important features for all outcomes were: CAP score, CAG repeats, accompanied or unaccompanied at clinic visit, tetrabenazine use, antipsychotics use and having severe cognitive impairment.

Unadjusted R^2^ measures were calculated for the RF models including CAG and CAP score only and compared with the model including all the features (Table 4). Using additional features with CAP and CAG can capture the variance of outcome by 17% more for cUHDRS and 15% more on average for the other outcomes. The slight improvement in model fit with CAP, CAG and age compared with the model built with the shared top 10 features could be due to the different cross-validation sets, and also the very high contributions of CAP and CAG to the model fit.

**Table 4:** Comparison of model performance with all the features (1), with the discovered top-ranking features (2) and with only established prognostic features (3 and 4).

	**Model**	**Outcome variable**				
		**cUHDRS**	**SDMT**	**TFC**	**SWR**	**TMS**
1	R^2^ with all the features	41%	28%	30%	26%	36%
2	R^2^ with the shared top 10 features^*^	31%	18%	20%	14%	24%
3	R^2^ with CAP, CAG and age at baseline	29%	19%	20%	16%	25%
4	R^2^ with CAP and CAG	24%	14%	15%	12%	20%
	Difference between 4 and 1	17%	14%	15%	14%	16%

* *Shared features among the individual top 10 important features of each outcome: CAP, CAG, accompanied or alone at visit, tetrabenazine use, antipsychotics use and cognitive impairment*.

*CAG, cytosine adenine guanine; CAP, CAG-age product; cUHDRS, composite Unified HD Rating Scale; SDMT, symbol digit modalities test; SWR, stroop word reading; TFC, total functional capacity; TMS, total motor score*.

## Discussion

This analysis used real-world data from the large Enroll-HD registry and a machine learning algorithm to identify novel predictors of HD progression with significant impact on the slope of clinical decline observed over a 2-year follow-up period. The two most important predictors identified were CAP score and CAG repeat length, in agreement with previous studies (7, 13). In addition, several strong predictors were identified that have either not been previously studied (being accompanied to a visit) or have had inconsistent effects in other studies (cognitive impairment, use of tetrabenazine or antipsychotics) (7, 17–19). The novel variables identified were predictive of progression over multiple clinical domains, measured by motor, cognitive and functional endpoints, as well as the composite endpoint. Using all predictors in addition to known prognostic variables improved the ability of the model to predict clinical outcomes (see video abstract in the Supplementary Materials).

Some of the features tested, which may have been expected to rank highly as prognostic variables based on previous studies in premanifest HD (i.e., prior to the onset of unequivocal motor symptoms)—including smoking, alcohol intake and body mass index (BMI) (20–22)—were not found to be important predictors of progression. These results may not be directly comparable to the current study, which was carried out in a manifest HD population. It is also known that self-reporting of smoking and alcohol use is unreliable in the general population, as revealed by studies using advances in DNA methylation measurement to assess substance use status (23). We found BMI to be a weak discriminating factor among patients with different values of change in outcome. A further potential explanation for the disparity between our findings and previous studies could be that the prognostic value of these variables may be dependent on disease stage. In the current study, the population was relatively progressed, and it is possible that other variables associated with the disease could outweigh environmental variables.

It should be noted that we used an RF algorithm with the setting that prevents bias in the ranking based on the data structure. Whilst it is still possible that a feature can rank highly due to collinearity with another feature that is a strong predictor of the outcome, this could be prevented by calculating the conditional importance of the features, which is computationally very complex (24).

Additionally, the observed associations are based on observational data and are therefore not indicative of causal relationships, due to measured and unmeasured potential confounding factors. For example, being accompanied to clinic visits may affect the clinical outcome scores measured by virtue of the companion's additional report which informs the clinical rating. It may also be because healthier participants are able to continually attend visits alone, whereas those who are on worse clinical trajectories need additional emotional or practical assistance (e.g., driving) to complete visits. Similarly, antipsychotics may be used to treat motor symptoms in HD, and therefore may be expected to reduce TMS without influencing overall disease progression. Cognitive outcome measures may also be related to variables including dementia and severe cognitive impairment. RF approaches have good performance in modelling complex, multidimensional disease-specific datasets (like Enroll-HD) (25). In this application, an RF approach was used to find novel associations (e.g., identify variables with predictive potential for disease progression), and does not imply causality (i.e., the aetiological role of the variable during disease progression) (26). Nevertheless, by virtue of the strength of the associations observed, some of these features may be important to control for in analyses of observational studies and may have implications for companion participation in interventional trials.

The current study focused on clinical variables only and did not include imaging or fluid biomarkers, which previous studies have suggested may be predictive of disease progression (7, 27, 28). This limitation was due to the nature of the currently available HD databases. In this study, we used the Enroll-HD database, which provides a sufficiently large sample size for the analysis but does not include imaging or biofluid data as part of the main study. Imaging databases such as PREDICT-HD (NCT00051324) are available, but do not provide the comprehensive range of clinical variables that is available in Enroll-HD, such as medication history. The available biofluid databases, such as the HD-CSF study (28, 29), are too small to be informative on the scale of the current analysis.

A further limitation is that the results described here are based on a selected cohort intended to reflect the inclusion criteria of ongoing clinical trials, and therefore may not be representative of the wider HD population, including younger patients (juvenile-onset HD), elderly patients (>65 years), late-stage patients (>Stage III) or premanifest patients. Further research is needed to determine the wider applicability of these results to these populations.

This study made use of a supervised RF regression model to identify putative and novel predictors of disease progression in HD. Identifying prognostic variables usually requires large sample sizes to optimise predictive accuracy, which may be a limitation for rare conditions such as HD. Since 2012, the Enroll-HD registry, which includes over 19,000 participants from 177 sites in 20 countries, has allowed a large, high-quality dataset to be available for researchers to advance the understanding of HD. The power of RF modelling is particularly relevant within the context of HD, where improved understanding of this multidomain disease and need for efficient trial design is evident.

To overcome known methodological limitations, RF approaches are being developed to harness the full potential of long-term registry data in clinical risk prediction and may, in future, accelerate disease risk and course prediction in HD. Given the dynamic nature of disease, the recently published RF Survival, Longitudinal and Multivariate model was developed to evaluate the temporal nature of variables (such as rate of change of variables) (30). Such approaches will further refine identification of clinically meaningful predictive variables not only for risk of disease progression as a static entity, but risk of disease progression over time and clinical course. Such temporal approaches will prove useful for future studies in HD, where disease course is highly variable.

In summary, the RF approach described here using the Enroll-HD dataset has identified novel prognostic variables which may be important candidates for statistical control in clinical trials and observational studies in HD.

## Methods

### Data Source

Data from the Enroll-HD database were used for this study. Enroll-HD is a global platform designed to facilitate clinical research in HD. Core variables are collected annually from all research participants as part of this multicentre, longitudinal, observational study. Data are monitored for quality and accuracy using a risk-based monitoring approach. All sites are required to obtain and maintain local ethical approval. The study began recruiting in 2012, and as of data released in 2018, includes over 19,000 total participants and more than 8,000 patients with manifest HD. The second version of the fourth periodic dataset release (PDS4 version 2.0) was used, which has a data cut-off date of 31 October 2018 and was made available in August 2019.

### Patient Population

The study population is purposely limited to individuals meeting typical criteria for clinical trials in manifest HD, using the filtering criteria shown in Table 1.

The primary population of interest was individuals with manifest HD aged 25–65 years, inclusive. Patients with juvenile-onset HD (age of first symptom onset at age <20 years) were excluded. Participants were required to have Independence Scale >70 at baseline and at least two subsequent annual visits with clinical information recorded. The rationale for these criteria is that the typical duration of clinical studies in this population is 2 years.

### Analytical Approach

A total of 102 prognostic variables (Table 5) were considered for each participant, including demographics, clinical characteristics, comorbidities, symptoms, as well as pharmacological and non-pharmacological treatments at baseline. The predicted variables are the estimated change of outcome measures over time. Estimated linear change was calculated for five outcome measures of HD which have known sensitivity to detect clinical change (change from baseline was measured out to 2 years): TFC, SWR, SDMT, TMS, and cUHDRS. The slope was estimated based on a linear mixed model with fixed and random intercept and slope respectively, and the individual-specific slope was computed as the sum of the random and fixed slope. Follow-up assessments up to 2 years that fell within a ± 90-day window around planned annual visits were included.

**Table 5:** List of candidate prognostic variables included in analyses.

Age	Marital status—separated	Nutrition—homeopathic
Age at diagnosis	Marital status—single	Nutrition—aromatherapies
Rater's judgement of initial major symptom—motor	Residence—rural	Non-pharmacological therapies—physical therapy
Rater's judgement of initial major symptom—cognitive	Residence—village	Non-pharmacological therapies—occupational therapy
Rater's judgement of initial major symptom—psychiatric	Residence—town	Non-pharmacological therapies—psychotherapy
Rater's judgement of initial major symptom—oculomotor	Residence—city	Non-pharmacological therapies—counselling
Rater's judgement of initial major symptom—other	Residence—unknown	Non-pharmacological therapies—speech therapy
Rater's judgement of initial major symptom—mixed	Region—Australasia	Non-pharmacological therapies—swallowing therapy
Rater's judgement of initial major symptom—unknown or missing	Region—Europe	Non-pharmacological therapies—music therapy
CAG repeats (affected allele)	Region—Latin America	Non-pharmacological therapies—relaxation therapy
CAG repeats (unaffected allele)	Region—North America	Non-pharmacological therapies—acupuncture
Baseline CAP score—age^*^(affected CAG repeats−33.66)	BMI	Accompanied to clinic visit^*^
Male sex	Comorbidities—renal	Unaccompanied to clinic visit^*^
Female sex	Comorbidities—gynaecological	Mix of accompanied and unaccompanied to clinic visit^*^
Race—black	Comorbidities—reproductive	History of irritability
Race—Native American	Comorbidities—dermatological	History of depression
Race—Asian	Comorbidities—musculoskeletal	History of violence/aggression
Race—Caucasian	Comorbidities—neurological	History of apathy
Race—Hispanic/Latin American	Comorbidities—metabolic	History of perseverative obsessive MH behaviours
Race—mixed	Comorbidities—psychiatric	History of psychosis (hallucinations MH or delusions)
Race—other	Comorbidities—ENT	Family history of psychotic illness in 1^st^ degree relative
Previous alcohol problems	Comorbidities—gastrointestinal	Significant cognitive impairment or dementia
Ever smoked	Comorbidities—allergy: immunological	History of motor symptoms MH compatible with HD
Currently smoke	Comorbidities—pulmonary	Drugs for anti-depression
Ever abused drugs	Comorbidities—ophthalmological	Lipid-modifying agents, plain and combinations
Current drug abuse	Comorbidities—cardiovascular	Thyroid therapies
Currently drink alcohol	Comorbidities—hepatobiliary	Antipsychotics use
Father affected by HD	Comorbidities—haematological/lymphatic	Anxiolytics, hypnotics and sedatives use
Mother affected by HD	Comorbidities—none	Analgesics use
Inheritance unknown	Comorbidities—other	Tetrabenazine use
Education—bachelor's degree or higher	Nutrition—vitamin	ACE inhibitors (plain and combination)
Education—post secondary but not university degree	Nutrition—herbs	Anti-epileptics use
Education—upper secondary or lower	Nutrition—teas	Protein pump inhibitors use
Marital status—married	Nutrition—other	Dopaminergic therapies use

*ACE, angiotensin-converting enzyme; CAG, cytosine adenine guanine; CAP, CAG-age product; ENT, ear, nose, throat; HD, Huntington's disease; MH, mental health*.

* *Accompanied/unaccompanied/mix of accompanied and unaccompanied to clinical visits were separated into a trichotomous variable. Accompanied means the patient did not come alone to all Enroll-HD study visits throughout follow-up (maximum three visits); Unaccompanied means they came alone to all visits; Mix of accompanied and unaccompanied means they sometimes came alone, but not always. This is the only candidate predictive variable based on some post-baseline data*.

An RF regression model with 1,000 trees was trained to rank the features on their ability to predict the estimated linear change of each clinical outcome. The model randomly selects a subset of 34 variables (one third of all available) for splitting at each node within each tree. The training was repeated 100 times, each time on a 75% random sample of the data. In each round, permutation importance of each feature for prediction of the outcome was calculated and used for the ranking of the features. The median of the rankings of these 100 models was used for the final ranking of the feature importance.

The R^2^ measure (the percentage of the slope variance that is explained by the model) was calculated for the following models predicting each outcome—a model trained with CAP score and CAG only, a model trained with CAP score, CAG and age, a model trained with the above-mentioned shared top 10 ranked features and a model trained with all features.

The analysis was done using R version 3.5.2, with lmer() from the lme4 package for the linear mixed-effects model, and Cforest() from the Party package for the RF regression model.

## Data Availability Statement

The data analysed in this study was obtained from Enroll-HD, https://enroll-hd.org/, the following licenses/restrictions apply: to access data you must be a researcher employed by a recognized academic institution, company or non-profit organisation and apply for an Enroll-HD access account. Requests to access these datasets should be directed to https://enroll-hd.org/for-researchers/become-a-qualified-researcher/.

## Code Availability

All the codes and algorithms for the analysis are available upon request.

## Author Contributions

NG contributed to the conception and design of the study, and acquisition and interpretation of data for the work. SS contributed to conception of the study, and acquisition and interpretation of data for the work. GP and PW contributed to the design of the study. JL contributed to the conception and design of the study. RH contributed to conception and design of the study, and acquisition and analysis of data for the work. All authors drafted or substantively revised a significant portion of the manuscript or figures, and approved the final version for submission.

## Conflict of Interest

NG, RH, SS, and GP are employees of F. Hoffmann-La Roche Ltd. JL is a paid Advisory Board member for F. Hoffmann-La Roche Ltd and uniQure biopharma B.V, and a paid consultant for Vaccinex Inc, Wave Life Sciences USA Inc, Genentech Inc and Triplet Inc. The remaining author declares that the research was conducted in the absence of any commercial or financial relationships that could be construed as a potential conflict of interest. The authors declare that this study received funding from F. Hoffmann-La Roche Ltd. The funder was involved in the study design, analysis, interpretation of data and the decision to submit it for publication.
